# *Rhodiola rosea* L. Attenuates Cigarette Smoke and Lipopolysaccharide-Induced COPD in Rats via Inflammation Inhibition and Antioxidant and Antifibrosis Pathways

**DOI:** 10.1155/2021/6103158

**Published:** 2021-03-02

**Authors:** Huanyue Cui, Xueying Liu, Jin Zhang, Ke Zhang, Dahong Yao, Shi Dong, Shushu Feng, Lu Yang, Yuyao Li, Hangyu Wang, Jian Huang, Jinhui Wang

**Affiliations:** ^1^Key Laboratory of Xinjiang Phytomedicine Resource and Utilization, Ministry of Education, College of Pharmacy, Shihezi University, Shihezi 832002, China; ^2^School of Pharmaceutical Sciences, Shenzhen University, Shenzhen 518060, China; ^3^School of Pharmaceutical Sciences, Shenzhen Technology University, Shenzhen 518060, China; ^4^Economic Forest Product Quality Inspection and Testing Center of the State Forestry Administration (Urumqi), Xinjiang Academy of Forestry, Urumqi 830000, China; ^5^Department of Medicinal Chemistry and Natural Medicine Chemistry (State-Province Key Laboratories of Biomedicine-Pharmaceutics of China), Harbin Medical University, Harbin 150081, China; ^6^Shenzhen Honghui Biopharmaceutical Co., Ltd., Shenzhen 518000, China

## Abstract

The root cause behind the development of chronic obstructive pulmonary disease (COPD) is cigarette smoke that induces the inflammation of the lung tissue and alveolar destruction. Long-term cigarette smoking can lead to deterioration in lung parenchymal function and cause structural changes in the lung, further resulting in pulmonary fibrosis. *Rhodiola rosea* L., a traditional medicinal perennial herb, is well known for its numerous pharmacological benefits, including anti-inflammation, antioxidant, antifatigue, antidepressive, and antifibrotic properties. Here, we evaluated the pharmacological effects and mechanisms of the *Rhodiola rosea* L. (RRL) macroporous resin extract on COPD caused by lipopolysaccharide (LPS) and cigarette smoke (CS) in rats. The RRL significantly improved the pathological structure of the lung tissue. Additionally, RRL decreased the infiltration of inflammatory cells and, subsequently, oxidative stress. Furthermore, the RNAseq assay indicated that RRL attenuated the CS and LPS-induced COPD via anti-inflammatory, antifibrotic, and antiapoptotic activities. Western blot analysis substantiated that the RRL resulted in upregulated levels of Nrf2 and HO-1 as well as downregulated levels of I*κ*B*α*, NF-*κ*B p65, *α*-SMA, and TGF-*β*1. Interestingly, the RRL could protect rats from CS and LPS-induced COPD by inhibiting the ERK1/2 and Smad3 signaling pathways and apoptosis. Thus, the RRL could attenuate CS and LPS-induced COPD through inflammation inhibition and antioxidant and antifibrosis pathways.

## 1. Introduction

Chronic obstructive pulmonary disease (COPD) is characterized by an increased chronic inflammatory response, resulting from the presence of toxic gases in the airway and lungs, resulting in an irreversible airflow obstruction [1]. The morbidity and mortality of COPD are still high to date, the prevalence of COPD in China has reached 8.6%, the number of patients suffering from COPD is nearly 99.9 million, and the annual mortality rate is as high as 1.28 million [2]. It adversely impacts the quality of life and is cost-intensive for the healthcare system [3]; however, its pathogenesis is not completely understood. Previous studies have indicated towards the involvement of pulmonary inflammation, oxidation imbalance, and protease-antiprotease imbalance in the development of COPD [4]. Studies have demonstrated that the release of reactive oxygen species (ROS) in patients with COPD mainly comes from cigarette smoke (CS), which contains high concentrations of oxidants that can directly induce lung inflammatory response and oxidative stress [5–7]. Oxidative stress can increase airway inflammation, which leads to progressive irreversible airflow limitation, which aggravates the development of COPD and eventually leads to bronchitis, emphysema, and pulmonary fibrosis [8]. Despite advancements in COPD therapy, there is an urgent need to discover effective drugs for COPD treatment.

*Rhodiola rosea* L.(RRL; family: Crassulaceae) is a traditional medicinal perennial herb, which is mainly distributed among the alpine gravel and rock gaps between Europe, North America, and Asia with high altitude, high cold, hypoxia, and a large temperature difference between day and night. It is a medicinal and food homologous plant with strong environmental adaptability and vitality and is widely used in the fields of medicine, food, and health products [9, 10]. Previous studies have isolated phenylethanol and glycosides (tyrosin, salidroside), phenylpropanins (rosavin, rosarin), flavonoid glycosides, cyanogenic glycosides, terpenoid polysaccharides, and amino acids from RRL [11]. However, the therapeutic effects of RRL are due to the flavonoids, phenylpropanoids, and organic acids present in its roots and rhizomes. RRL possesses anti-inflammatory [12], antioxidant [13], antifatigue, antidepressive [14], antifibrotic [9], antiallergic, anticancer [15], antiapoptotic [16], and several other pharmacological activities. RRL has recently been thought to play an important role in improving lung injuries. It has been reported that salidroside can mitigate lipopolysaccharide or paraquat-induced acute lung injuries in animals [17, 18]. Researchers have studied the effect of RRL on pulmonary fibrosis in rats; the results showed that RRL could significantly reduce the pulmonary inflammatory response and oxidative stress and inhibit the development of pulmonary fibrosis [9]. These earlier studies mainly focused on the antifibrosis, antioxidation, and anti-inflammation effects of RRL in lung tissues or cells. However, there is a scarcity of reports on the pharmacological effects and mechanism of action of RRL in COPD.

Lipopolysaccharide (LPS) and cigarette smoke (CS) are the preferred stimulators in COPD research [19]. CS is a mixture of more than 5,000 chemical substances, including reactive oxygen species (ROS) and oxygen-derived metabolites which play an important role in maintaining homeostasis, cell signaling, and antibacterial activity. However, the excessive accumulation of ROS in the body will lead to harmful modification of lipid, protein, and DNA [20, 21]. LPS is a component of the cell wall of gram-negative bacteria present in atmospheric pollutants. When bacteria invade an organism, the lipopolysaccharide on the cell wall stimulates neutrophils, macrophages, and epithelial cells to release inflammation factors and eventually induce inflammation in lung tissue and airways [22]. Here, we explored the therapeutic effects and mechanism of action of RRL in COPD by establishing a rat model of oxidative stress and severe inflammation by smoke inhalation and LPS intratracheal instillation. The results indicated that RRL could significantly ameliorate the CS and LPS-induced COPD through its antioxidative, anti-inflammatory, and antifibrotic properties. These results support the further development of RRL as a therapeutic agent for the treatment of COPD.

## 2. Materials and Methods

### 2.1. Animals

Sprague Dawley rats (male; 4–6 weeks; 180 ± 10 g) of SPF (specific pathogen free) were procured from the Laboratory Animal Center of the Xinjiang Medical University, with the license number SCXK (Xin) 2016-0001. The rats were placed in controlled environmental conditions with a 12-hour light/dark cycle. During the experiment, the rats were free to obtain water and food. Animal welfare and the experimental procedures were consistent with the Ethical Regulations of the Care and Use of Laboratory Animals of Shihezi University, and all animal experiments were performed with the approval and under the guidelines of the Animal Experimental Ethics Committee of the First Affiliated Hospital of Medical College, Shihezi University (A2019-055-01, 05 March 2019).

### 2.2. Materials

We collected the root and rhizome samples of RRL at Tacheng, Xinjiang, China, which were identified by Yong Tan (Shihezi University, China). Lipopolysaccharides (LPS) (L8880) and 3-(4,5-dimethylthiazol-2-yl)-2,5-diphenyltetrazolium bromide (MTT) (M8180) were procured from Solarbio Science and Technology Co., Ltd. (Beijing, China). Dexamethasone (Dex) tablets were bought from Zhengzhou Zhuofeng Pharmaceutical Co., Ltd. (Zhengzhou, China). Enzyme-linked immunosorbent assay (ELISA) kits for rat IL-6 (F15870), TNF-*α* (F16960), and IL-8 (F15880) were purchased from Shanghai Westang Bio-Tech Co., Ltd. (Shanghai, China). The BCA total protein assay kit (A045-3), Masson's trichrome stain kit (D026), SOD (A001-3), MDA (A003-1), Wright's-Giemsa stain kit (D011-1), and GSH-Px (A005) were all purchased from Nanjing Jiancheng Bioengineering Institute (Nanjing, China). NF-*κ*Bp65 antibody (BM3940), I*κ*B*α* antibody (BM3932), TGF-*β*1 antibody (BA0290), *α*-SMA antibody (BM3902), Nrf2 antibody (PB9290), GAPDH antibody (BM1985), HO-1 antibody (M00253-2), Ras antibody (BM4940), Raf antibody (BM4108), p-Raf antibody (BM4708), ERK1/2 antibody (BM4326), p-ERK1/2 antibody (BM5446), Bax antibody (BM3964), Bcl-2 antibody (BM4241), and Smad3 antibody (BA4559) were all obtained from Wuhan Boster Biological Engineering Co., Ltd. (Wuhan, China). *β*-Actin antibody (16A00205) was from ZSGB-BIO (Beijing, China). Dimethyl sulfoxide (DMSO) (D8370) was from Sigma-Aldrich (United States of America). Dulbecco's Modified Eagle Medium Nutrient Mixture F-12 (Ham) (DMEM/F12) was from Thermo Fisher Biochemicals (Beijing) Co., Ltd. (Beijing, China). Fetal bovine serum was from Zhejiang Tianhang Biological Technology Co., Ltd. (Zhejiang, China).

### 2.3. Preparation of *Rhodiola rosea* L. Macroporous Resin Extract

The dried rhizome of RRL (10.0 kg) was heated and refluxed 3 times (3.0 h each time) with 95% ethanol (solvent and sample ratio was 10 : 1, *v*/*w*). The solutions were collected and concentrated to dryness at 50°C using a rotary evaporator device to obtain 1.588 kg of extracts. The 1.588 kg of extracts was purified with HPD100 macroporous resin and then eluted with 70% ethanol. The collected elutions were concentrated and dried using a rotary evaporator device, and 650 g RRL macroporous resin extract was stored at 4°C for future research.

### 2.4. High Performance Liquid Chromatography

The RRL macroporous resin extract was dissolved in methanol and filtered using a polyvinylidenefluoride (PVDF) filter (0.2 *μ*m microspin). The phytochemical characterization was done using an HPLC system (Waters 2695) equipped with a Sumfire C18 column having the following dimensions: 5 *μ*m, 4.6 × 250 mm. The sample was eluted using a mobile phase containing (A) 0.1% phosphate in water and (B) methanol with the following step gradient pattern at 1.0 mL/min (35°C): 0–18 min, 4–10% B; 18–45 min, 10% B; 45–55 min, 10–45% B; 55–65 min, 45%–95% B; and 65–75 min, 95% B. Then, the column was reconditioned to its initial state for 10 min. We measured the absorbance of the eluted fractions at 210 nm.

### 2.5. Cell Culture

Human type II lung epithelial cell line A549 was obtained with Shanghai Cell Bank. The cells were cultured in DMEM/F12, supplemented with 10% fetal bovine serum. They were incubated at 37°C in a humidified atmosphere of 5% CO_2._ After reaching 80% confluence, the cells were subcultured for subsequent experiments.

### 2.6. MTT Assay

MTT colorimetric method is a method for detecting cell viability and growth status. A549 cells were cultured for 24 h in 96-well plates before adding RRL (5, 10, 20, 40, and 80 *μ*mol/L) or H_2_O_2_ (50, 100, 200, 400, and 800 *μ*mol/L) 100 *μ*L per well and incubated at 37°C for 24 hours to detect the effect of RRL or H_2_O_2_ on cell viability. A549 cells were cultured for 24 h in 96-well plates before adding RRL (5, 10, and 20 *μ*mol/L) or H_2_O_2_ (100 *μ*mol/L) in the positive control group 100 *μ*L per well and incubated at 37°C for 8, 12, and 24 hours to detect the protective effect of RRL on H_2_O_2_ damaged cells. All control groups do not add RRL or H_2_O_2_. Set 5 duplicate secondary holes for each dose. They were then incubated for 24 h at 37°C in a humidified atmosphere containing 5% CO_2_. Each well with 10 *μ*L MTT solution (5 mg/mL) was further subjected to cultivation for another 4 h. Following the culture, the supernatant was carefully discarded, and 150 *μ*L of dimethyl sulfoxide was added to each well. The suspension was oscillated on a shaker for 10 minutes, and the crystals were fully dissolved. Then, the absorbance of each well was measured at 490 nm using a thermo (3001) multifunctional microplate reader. The cell viability was calculated as OD for the treatment group/OD for the (positive) control group.

### 2.7. Intracellular Reduced Glutathione (GSH) Assay

CMFDA, a chloromethyl derivative of fluorescein diacetate (fluorescein diacetate, FDA), is nonfluorescent and passively accumulated in the cells because of its lipophilic property. Inside the cells, acetate residues are cleaved off by intracellular esterases, releasing the fluorescent and cell-impermeable product 5-chloromethyl fluorescein (CMF). 5-Chloromethyl fluorescein can emit green fluorescence and use its chloromethyl and glutathione in intracellular proteins and peptides to form adducts under the action of glutathione mercaptotransferase, which can be well preserved in the cell. A549 cells were cultured for 24 h in 96-well plates before adding RRL (5, 10, 20, 40, and 80 *μ*mol/L) 100 *μ*L per well and incubated at 37°C for 24 hours. All control groups do not add RRL or H_2_O_2_. 100 *μ*L H_2_O_2_ (100 *μ*mol/L) was added in the positive control group per well. Set 5 duplicate secondary holes for each dose. When the cell culture was finished, the culture plate was removed and 100 *μ*L CMFDA probes (5 *μ*mol/L) were added in each hole. Then, the absorbance of each well was measured at Ex = 492 nm and EmEx = 517 nm using a thermo (3001) multifunctional microplate reader after washing with DMEM/F12 incomplete medium three times. The cell viability was calculated as OD for the treatment group/OD for the (positive) control group. A549 cells were cultured for 24 h in 96-well plates before adding RRL (5, 10, 20, 40, and 80 *μ*mol/L) 1000 *μ*L per well and incubated at 37°C for 24 hours. All control groups do not add RRL or H_2_O_2_. 100 *μ*L H_2_O_2_ (100 *μ*mol/L) was added in the positive control group per well. Set 5 duplicate secondary holes for each dose. When the cell culture was finished, the culture plate was removed and 1000 *μ*L CMFDA probes (5 *μ*mol/L) were added in each hole. The results were recorded by after washing with DMEM/F12 incomplete medium three times. The results were recorded by Zeiss positive fluorescence microscope (MIC00266).

### 2.8. Animal Treatment

Sixty rats were randomly assigned to the following groups: the control group, Dex (1 mg/kg) group, model group, and RRL (200, 400, and 800 mg/kg) treatment groups. On day 1 and day 15 of the experiment, each rat in the model group and the treatment group was slowly injected with 200 *μ*g LPS into the trachea, while the control group rats were injected with an equivalent amount of saline into the trachea, and then the rats were placed vertically and shaken to distribute LPS evenly in the lungs. On the remaining days, the model group and the treatment group rats were placed in a homemade smokebox (60 cm^*∗*^35 cm^*∗*^30 cm), made of plexiglass, with a fan on the right side, and the rat was passively smoked in the smokebox. Both the model group and the treatment group rats were given ten cigarettes (tar, 25 mg; CO, 13 mg; nicotine, 1.1 mg) (Hongqi Canal® Filter Tip Cigarette, Henan Tobacco Industry, Zhengzhou, China) per group and passively smoked twice a day for 30 minutes each time. On the basis of model establishment, Dex group was given Dex (1 mg/kg), and RRL treatment group was given RRL (200, 400, 800 mg/kg). During the experiment, the weight of the rats was recorded daily, and the conditions of eating and drinking water, mental state, appearance changes, and feces were observed. On day 31 of the experiment, the rats were dissected, and lung tissue samples and serum along with bronchoalveolar lavage fluid (BALF) were collected.

### 2.9. Organ Coefficient and Histological Analysis

After removing the whole lung, immediately the wet weight is weighed to calculate the lung organ coefficient. The left lung was weighed immediately after removal (wet weight) and again after drying in an oven at 80°C for 48 h (dry weight) to calculate the lung wet/dry weight ratio. After fixing the lung tissue specimens in 10% formalin, they were dehydrated in ethyl alcohol and finally sectioned after embedding in paraffin. Next, the specimens were deparaffinized in xylene and rehydrated using gradient alcohol. The sections were treated with Masson's trichrome stain and hematoxylin and eosin (H&E) stain. The sections were evaluated blindly by medical pathologists. The alveolitis and fibrosis scoring criteria were as follows: no alveolitis or fibrosis, 0 points; mild alveolitis or fibrosis, range of lesion <20% of the lung, 1 point; moderate alveolitis or fibrosis, range of lesion involving 20%–50% of the lung, 2 points; severe alveolitis or fibrosis, range of lesion involving more than 50% of the lung, 3 points. Six microscopic fields were examined at a magnification of 400x and the collagen areas in the images were analyzed by used Image-Pro Plus professional image analysis software system [23].

### 2.10. Collection of BALF and Cell Counting

BALF was extracted by slowly instilling 0.5 mL cold PBS, and the process was repeated thrice. BALF (1.5 mL) was collected for the follow-up study. The collected BALF was centrifuged at 3000 rpm for 10 min. The pellet was resuspended in 50 *μ*L of cold PBS. Next, from this cell suspension, 10 *μ*L was used for cell counting using a hemocytometer, and 30 *μ*L was used for preparing cell smears, which were stained with Wright–Giemsa stain. The supernatant was used for cytokine analysis. An optical microscope was used to distinguish the different types of cells.

### 2.11. Assessment of Indices for Oxidative Stress in Serum and Lung Tissue

Commercial kits were used to evaluate the enzymatic activities of superoxide dismutase (SOD), glutathione peroxidase (GSH-Px), and concentration of malondialdehyde (MDA) in the serum and lung tissue. SOD belongs to the family of metalloproteinases. It plays an important role in protecting cells from oxidative damage by converting O^2−^ into H_2_O_2_ and maintaining the balance of oxygen free radicals in the body [24]. MDA is one of the end products of lipid peroxidation in the cell membrane; its content can reflect the degree of lipid peroxidation and indirectly reflect the degree of cell damage.

### 2.12. Immunohistochemistry

After dewaxing the lung specimens with xylene, the antigen was extracted using a microwave in citrate buffer (pH 6.0). Next, 3% hydrogen peroxide was used for blocking the endogenous peroxidase (10 min). After washing with PBS, the specimens were kept in overnight incubation with TGF-*β*1 (1 : 200), NF-*κ*B p65 (1 : 10), Nrf2 (1 : 200), and I*κ*B*α* (1 : 20) primary antibodies at 4°C. Next, the specimens were incubated with goat anti-rabbit or anti-mouse secondary antibodies at 37°C for 30 min. The specimens were stained with diaminobenzidine (DAB) and counterstained with hematoxylin. Finally, the samples were dehydrated to transparency and sealed with neutral balsam. Image-Pro Plus 6.0 was used to analyze the specimens.

### 2.13. Western Blot

We used the nuclear protein extraction kit to partially extract lung protein, while the other part was homogenized with a lysis buffer and centrifuged for 10 min at 4°C. The BCA protein assay kit was used to determine the protein concentration in the supernatant. First, SDS-PAGE was used to separate the protein molecules based on their molecular weight. Next, the protein was transferred onto the PVDF membranes, which were blocked using 5% skimmed milk. The membranes were kept in overnight incubation (4°C) with I*κ*B*α* (1 : 300), NF-*κ*B p65 (1 : 500), p- I*κ*B*α* (1 : 1000), HO-1 (1 : 500), Nrf2 (1 : 400), *α*-SMA (1 : 1000), TGF-*β*1 (1 : 800), Raf (1 : 200), Ras (1 : 300), p-Raf (1 : 200), ERK1/2 (1 : 200), Bax (1 : 500), p-ERK1/2 (1 : 200), Bcl-2 (1 : 500), and Smad3 (1 : 300) primary antibodies. Then, the membranes were incubated with horseradish peroxidase-conjugated secondary antibody, followed by treatment with ECL chemiluminescence reagent. Image-Pro Plus 6.0 was used to analyze the bands. *β*-Actin or GAPDH was used as the loading control.

### 2.14. Statistical Analyses

One-way ANOVA, followed by the Tukey post-hoc test, was performed to evaluate the statistical significance. Data were analyzed using SPSS v17.0 and presented as mean ± standard deviation (SD). Also, *P* value <0.05 was considered as statically significant.

## 3. Results

### 3.1. HPLC-DAD Analysis of the RRL Macroporous Resin Extract

**Figure 1:**
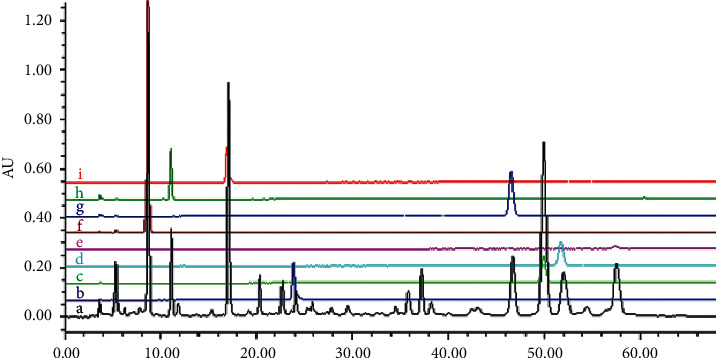
HPLC-DAD chromatogram (210 nm) of the RRL. a, RRL; b, benzyl-*β*-D-glucopyranoside; c, rosavin; d, rosin; e, rosiridin; f, gallic acid; g, rosarin; h, rhodiocyanoside A; i, salidroside.

First, we characterized the main chemical constituents of the RRL using HPLC-DAD. All calibration curves showed a good linear regression (*γ* ≥ 0.990), indicating that the method is accurate and sensitive to the quantitative evaluation of the main chemical constituents of RRL. Unequivocal identification was attained using phytochemical standards, such as benzyl-*β*-D-glucopyranoside, rosavin, rosin, rosiridin, gallic acid, rosarin, rhodiocyanoside A, and salidroside (Table 1, Figure 1).

### 3.2. The Effect of RRL on the Respiratory Improvement and Attenuation Histopathological Alterations Induced by CS and LPS

**Figure 2:**
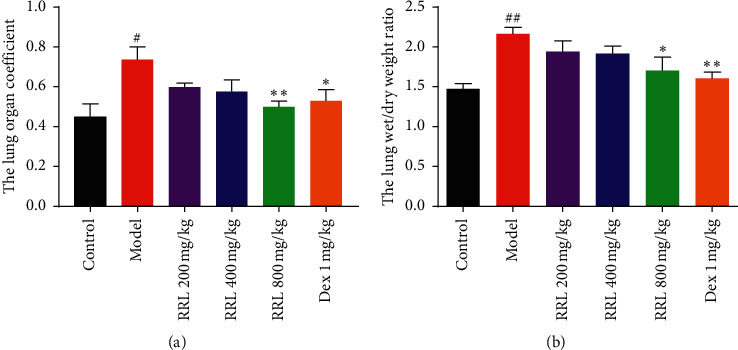
The effect of RRL on the respiratory improvement of CS and LPS-induced COPD in rats. (a) The lung organ coefficient, (b) the lung wet/dry weight ratio. Values are expressed as the mean ± SD; ^#^*P* < 0.05 vs. the control group;^##^*P* < 0.01 vs. the control group; ^*∗*^*P* < 0.05 and ^*∗∗*^*P* < 0.01 vs. the model group.

**Figure 3:**
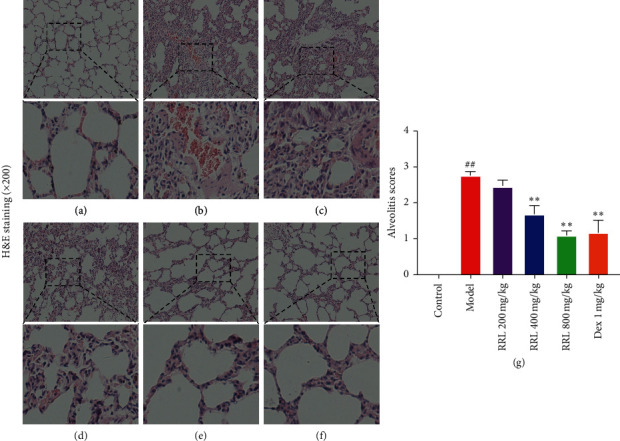
Effect of RRL on the pathomorphology of CS and LPS-induced COPD in rats (H&E staining). (a) Control group. (b) Model group. (c) RRL 200 mg/kg. (d) RRL 400 mg/kg. (e) RRL 800 mg/kg. (f) Dex 1 mg/kg. (g) Effect of RRL on the degree of alveolitis of CS and LPS-induced COPD in rats (*n* = 6, $\bar{X}$ ± *S*); ^##^*P* < 0.01 vs. the control group; ^*∗∗*^*P* < 0.01 vs. the model group.

The lung organ coefficient and the lung wet/dry weight ratio is to investigate the effect of RRL on the respiratory improvement. The results indicated that in the model group, the lung organ coefficient and the lung wet/dry weight ratio increased significantly (*P* < 0.05, *P* < 0.01; Table 2 and Figure 2), indicating that the permeability of alveolar capillaries increased and the fluid in the lungs leaked. The RRL (400 and 800 mg/kg) group and the Dex (1 mg/kg) group showed considerably lower levels of the lung body mass ratio and lung water content compared with the model group, indicating that the lung ventilation function and ventilation function of rats were improved and that RRL has a protective effect on the respiratory improvement. The lung tissue specimens were stained using H&E stain to investigate whether the RRL could alleviate CS and LPS-induced pulmonary histopathological alterations. The results indicated that in the control group, the lung tissues were intact without the thickening of the alveolar septum (Figure 3(a)), while that of model group had significant pathological changes compared to the control group, including severe destruction of the alveolar structure, and significantly thicker alveolar septum, enlarged alveolar cavity, along with a significant amount of infiltration of the inflammatory cell (*P* < 0.01; Figure 3(b)). Dose-dependent treatment with the RRL significantly attenuated CS and LPS-induced lung histopathological alterations compared with the control group (Figures 3(c)–3(e)). Interestingly, the RRL (800 mg/kg) group almost reversed the pathological alterations in the lung tissue.

**Figure 4:**
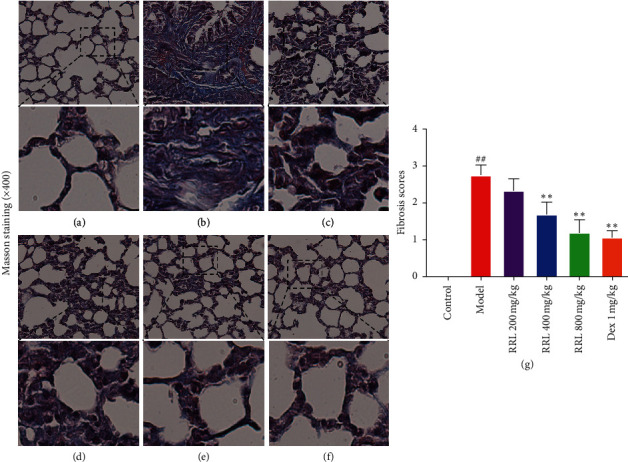
Effect of RRL on pathomorphology of CS and LPS-induced COPD in rats (Masson's trichrome staining). (a) Control group. (b) Model group. (c) RRL 200 mg/kg. (d) RRL 400 mg/kg. (e) RRL 800 mg/kg. (f) Dex 1 mg/kg. (g) Effect of RRL on the degree of fibrosis of CS and LPS-induced COPD in rats (*n* = 6, $\bar{X}$ ± *S*); ^##^*P* < 0.01 vs. the control group; ^*∗∗*^*P* < 0.01 vs. the model group.

Similarly, in the control group, we observed intact structure of the lung tissue (Masson's trichrome staining (Figure 4(a)). While in the model group, the alveolar structure was severely damaged, the parenchymal collagen deposition was greater, and there were considerably higher number of collagen fibers in the pulmonary interstitial and alveolar septum (*P* < 0.01; Figure 4(b). The RRL (200, 400, and 800 mg/kg) groups and the Dex (1 mg/kg) group showed a dose-dependent improvement in CS and LPS-induced pulmonary fibrosis (Figures 4(c)–4(f)). Thus, the RRL could be an effective therapeutic agent for CS and LPS-induced pulmonary fibrosis.

### 3.3. The Effect of RRL and H_2_O_2_ on Cell Viability and the Protective Effect of RRL on H_2_O_2_ Damaged Cells

**Figure 5:**
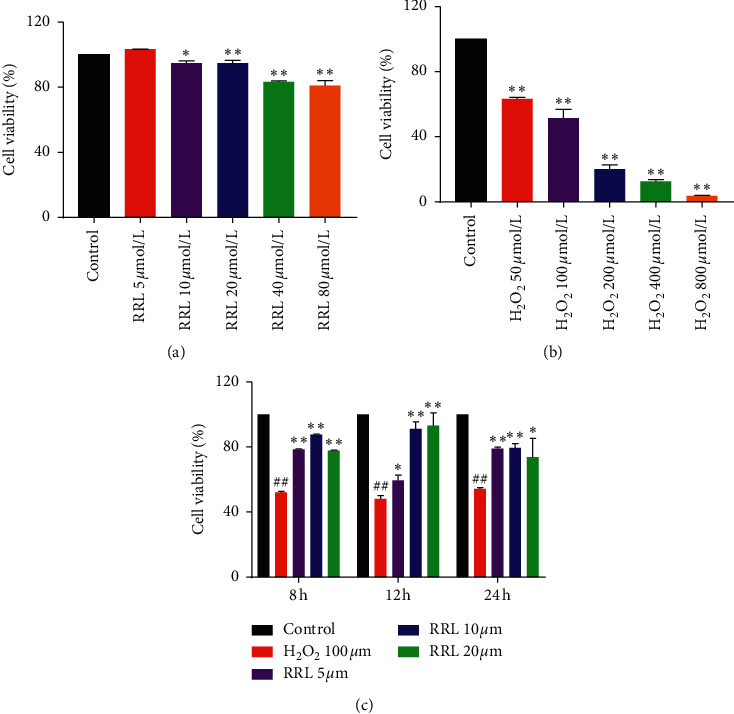
The effect of RRL and H_2_O_2_ on cell viability and the protective effect of RRL on H_2_O_2_ damaged cells. (a) The effect of RRL on cell viability. (b) The effect of H_2_O_2_ on cell viability. (c) The protective effect of RRL on H_2_O_2_ damaged cells. Values are expressed as the mean ± SD; ^##^*P* < 0.01 vs. the control group; ^*∗*^*P* < 0.05 and ^*∗∗*^*P* < 0.01 vs. the model group.

MTT assay was used to determine cell viability after treatment with RRL and H_2_O_2_. The data of MTT assay for RRL (Figure 5(a)) indicated cell viability is approximately 80% at the highest concentration (80 *μ*mol/L). When the RRL concentration is less than 20 *μ*mol/L (used to discuss the protective effect of RRL on H_2_O_2_ damage to A549 cells), the cell viability reaches more than 90%. The data of MTT assay for H_2_O_2_ (Figure 5(b)) indicated cell viability is inhibited in a concentration-dependent manner. When the H_2_O_2_ concentration is 100 *μ*mol/L, the cell viability is approximately 50% as a model of A549 cells damage. The protective effect of RRL on H_2_O_2_ damaged A549 cells by MTT assay (Figure 5(c)) indicated cell viability significantly (*P* < 0.05) increased at 5 *μ*m–20 *μ*m concentrations after 8 h, 12 h, and 24 h compared with H_2_O_2_ group. Interestingly, cell viability at 20 *μ*m was lower than at 10 *μ*m at 8 h and 24 h on contrary to 12 h. Thus, the RRL could substantially enhance the antioxidant levels to attenuate the H_2_O_2_-induced A549 cell injury.

### 3.4. The Effect of RRL on GSH Content in H_2_O_2_-Induced A549 Cell Injury

**Figure 6:**
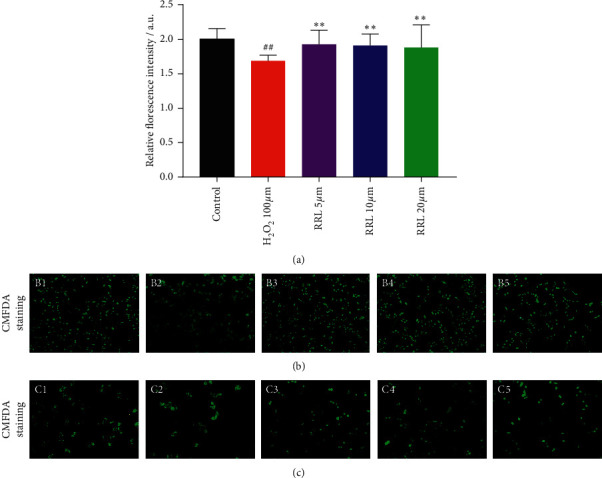
The effect of RRL on GSH content in H_2_O_2_-induced A549 cell injury. (a) Results obtained with thermo 3001 multifunctional microplate reader. (b) Results obtained with a upright fluorescence microscope 10 times larger and B1–B5 are control, H_2_O_2_ (100 *μ*mol/L), RRL (5 *μ*mol/L), RRL (10 *μ*mol/L), and RRL (20 *μ*mol/L). (c) Results obtained with a upright fluorescence microscope 20 times larger and C1–C5 are control, H_2_O_2_ (100 *μ*mol/L), RRL (5 *μ*mol/L), RRL (10 *μ*mol/L), and RRL (20 *μ*mol/L). Values are expressed as the mean ± SD; ^##^*P* < 0.01 vs. the control group; ^*∗∗*^*P* < 0.01 vs. the model group.

We determined the GSH content using the CMFDA method to investigate whether the RRL could increase the antioxidant levels in H_2_O_2-_induced A549 cell injury. There was a considerable decrease in the levels of GSH in the H_2_O_2_ group than the control group (*P* < 0.01; Figures 6(a)–6(c)); however, a significant enhancement in the RRL group (5, 10, 20, 40, and 80 *μ*mol/L) compared to the H_2_O_2_ group was observed. Thus, the RRL could substantially enhance the GSH content to attenuate the H_2_O_2_-induced A549 cell injury.

### 3.5. Effects of RRL on Inflammatory Cell Count in BALF

**Figure 7:**
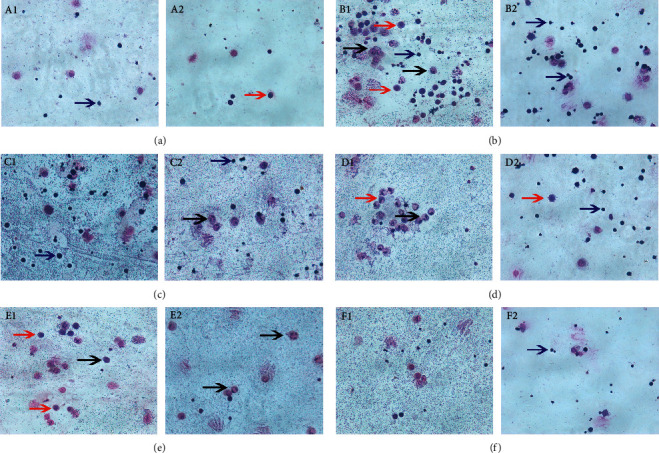
Wright–Giemsa staining in BALF of rats in each group. (a) A1–A2, control group. (b) B1–B2, model group. (c) C1–C2 RRL 200 mg/kg. (d) D1–D2, RRL 400 mg/kg. (e) E1–E2, RRL 800 mg/kg. (f) F1–F2, DEX 1 mg/kg. Red arrow, macrophages. Blue arrow, lymphocytes. Black arrow, neutrophils.

**Figure 8:**
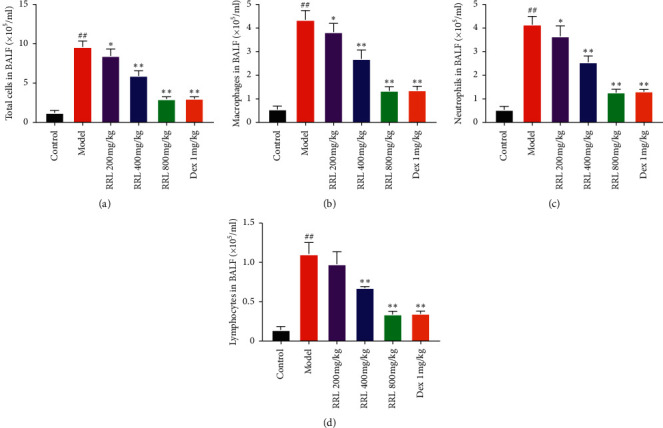
Cellular count and categorization in the BALF of rats. (a) The total number of cells in BALF. (b) The number of macrophages in BALF. (c) The number of neutrophils in BALF. (d) The number of lymphocytes in BALF. Values are expressed as the mean ± SD; ^##^*P* < 0.01 vs. the control group; ^*∗*^*P* < 0.05 and ^*∗∗*^*P* < 0.01 vs. the model group.

After Wright–Giemsa staining of BALF cell smears of rats in each group, 3 types of cells were found: macrophages, neutrophils, and lymphocytes (Figures 7 and 8). Next, we classified and counted the cells in BALF to study the effects of the RRL on the infiltration of the inflammatory cells in the lungs. The total number of cells in the model group were substantially higher than the control group (*P* < 0.01; Figure 7(a)). Also, the RRL (200, 400, and 800 mg/kg)-treated cells showed a dose-dependent decrease in their number, which indicated that RRL was effective in reducing the total number of cells in BALF. Additionally, there were considerably higher number of macrophages, neutrophils, and lymphocytes in BALF in the model group than in the control group (*P* < 0.01; Figures 7(b)–7(d)). We also observed a substantial decrease in these cells in the RRL (800 mg/kg) group and the Dex (1 mg/kg) group (*P* < 0.01; Figures 7(b)–7(d)). Thus, the RRL significantly inhibited the synthesis of inflammatory cells in BALF.

### 3.6. Effect of RRL on IL-6, IL-8, and TNF-*α* in CS and LPS-Induced COPD in Rats

**Figure 9:**
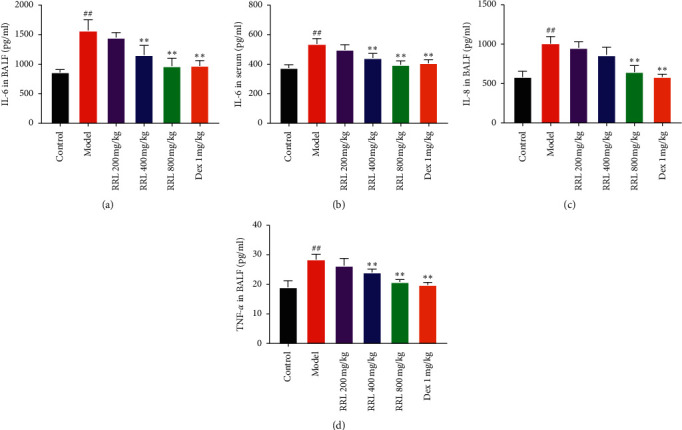
Effect of RRL on the levels of IL-6, IL-8, and TNF-*α* in CS and LPS-induced COPD in rats (*n* = 6, *X* ± *S*). (a) ELISA kits revealed the level of IL-6 in BALF. (b) ELISA kits revealed the level of IL-6 in serum. (c) ELISA kits revealed the level of IL-8 in BALF. (d) ELISA kits revealed the level of TNF-*α* in BALF. Values are expressed as the mean ± SD; ^##^*P* < 0.01 vs. the control group; ^*∗*^*P* < 0.05 and ^*∗∗*^*P* < 0.01 vs. the model group.

To further investigate the effect of RRL on pulmonary inflammatory response in COPD model rats, we evaluated the expression of IL-6 in serum and BALF of rats and of TNF-*α* and IL-8 in BALF of rats. There was an upregulated expression of IL-6 in the serum and BALF of rats in the model group than the control group (*P* < 0.01; Figures 9(a) and 9(b)), while its expression was downregulated in the RRL (400 and 800 mg/kg) group and the Dex (1 mg/kg) group (*P* < 0.01; Figures 9(a) and 9(b)). Also, the levels of IL-8 and TNF-*α* in BALF were upregulated in the model group than the control group (*P* < 0.01; Figures 9(c) and 9(d)) and were downregulated in the RRL (800 mg/kg) group and the Dex (1 mg/kg) group than the model group (*P* < 0.01; Figures 9(c) and 9(d)). Thus, the RRL decreased the levels of inflammatory factors in the serum and BALF of COPD rats.

### 3.7. Effect of RRL Lung Tissue SOD, GSH-Px, and MDA in CS and LPS-Induced COPD

**Figure 10:**
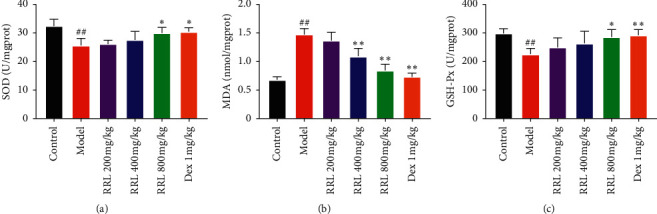
Effect of the RRL on GSH-Px, SOD, and MDA activity in CS and LPS-induced COPD in rats (*n* = 6, *X* ± *S*). (a) The activity of SOD in lung tissue. (b) The content of MDA in lung tissue. (c) The activity of GSH-Px in lung tissue. Values are expressed as the mean ± SD; ^##^*P* < 0.01 vs. the control group; ^*∗*^*P* < 0.05 and ^*∗∗*^*P* < 0.01 vs. the model group.

We determined the oxidation products, such as MDA, SOD, and GSH-Px, to investigate whether the RRL could increase the antioxidant levels during CS and LPS-induced COPD in rats. There was a considerable enhancement in the levels of MDA in the model group than the control group (*P* < 0.01; Figure 10(b)); however, a significant decrease in the SOD and GSH-Px catalytic activities was observed (*P* < 0.01; Figures 10(a) and 10(c)). The RRL (400 and 800 mg/kg) group and the Dex (1 mg/kg) group showed considerably lower levels of MDA and higher GSH-Px and SOD catalytic activities compared with the model group (*P* < 0.05, *P* < 0.01; Figure 10). Thus, the RRL could substantially enhance the antioxidant levels, in turn, attenuating the lung damage caused by oxidative stress.

### 3.8. Effect of the RRL on the Expression of NF-*κ*B p65, TGF-*β*1, I*κ*B*α,* and Nrf2 in CS and LPS-Induced COPD in Rats

**Figure 11:**
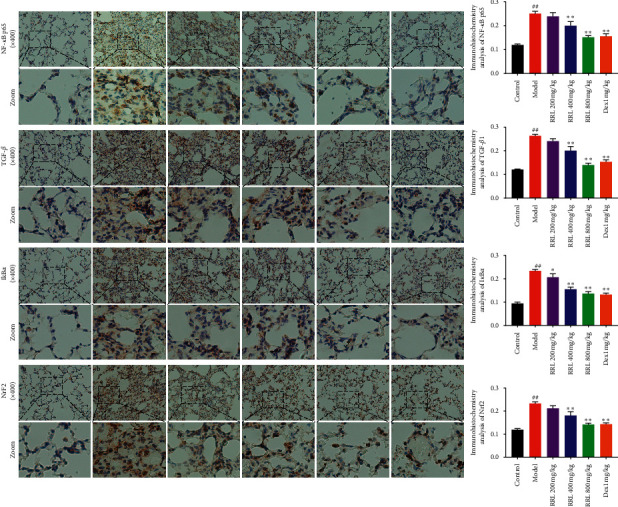
The effect of RRL on TGF-*β*1, NF*κ*B p65, I*κ*B*α,* and Nrf2 levels in CS and LPS-induced COPD in rats. a, control group; b, model group; c, RRL 200 mg/kg; d, RRL 400 mg/kg; e, RRL 800 mg/kg; f, Dex 1 mg/kg. Values are expressed as the mean ± SD (*n* = 3); ^##^*P* < 0.01 vs. the control group; ^*∗*^*P* < 0.05 and ^*∗∗*^*P* < 0.01 vs. the model group.

NF-*κ*B p65 and I*κ*B*α* play an important role in the development of lung inflammation [25], and TGF-*β*1 is a crucial fibrogenic cytokine, which is highly expressed in lung tissue with fibrosis [26]; Nrf2 is the most important antioxidant factor in organisms [27]. Therefore, we used immunohistochemistry to study the levels of NF*κ*B p65, I*κ*B*α*, TGF-*β*1, and Nrf2 in lung tissue specimens, which were upregulated in the model group compared to the control group (*P* < 0.01; Figure 11). Furthermore, the RRL (400 and 800 mg/kg) and Dex (1 mg/kg) exhibited their therapeutic effects by inducing a dose-dependent downregulation of the levels of NF*κ*B p65, I*κ*B*α*, TGF-*β*1, and Nrf2. Thus, the RRL significantly inhibited the inflammation, oxidative stress, and fibrosis of lung tissue in CS and LPS-induced COPD in rats.

### 3.9. RNAseq Expression/Pathways

**Figure 12:**
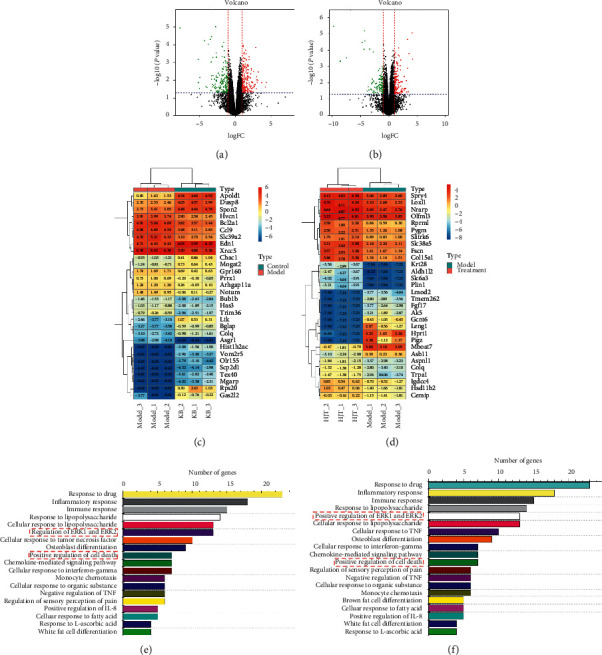
RNAseq analysis revealed that RRL could alleviate COPD via inflammatory and ERK signaling. (a) Volcano plot showed the expression profiling changes of control and model groups. (b) Volcano plot showed the expression profiling changes of model and RRL groups. (c) Heat map showed the expression of genes that were up- or downregulated (control and model groups). (d) Heat map showed the expression of genes that were up- or downregulated (model and RRL groups). (e) Top signaling pathways affected by model group gene expression changes. (f) Top signaling pathways affected by RRL treatment gene expression changes.

We performed RNAseq on the lung tissue specimens, collected from the control group, model group, and treatment group (RRL 800 mg/kg) to understand the entire transcriptional changes stimulated by RRL in COPD-related processes. Our results showed that 459 genes showed significantly differential expression after exposure to CS (fold change value >2, adjust *P* < 0.05), of which 238 genes were upregulated, and 221 genes were downregulated in the model group compared to the control group (Figure 12(a)). The heatmap shows the top 30 genes (Figure 12(c)). These differential genes were annotated using gene ontology (GO), which revealed that they were closely related to multiple biological processes, including drug-response, inflammatory response, immune response, cell death, etc. (Figure 12(e)). The inflammatory response was identified as an important change that occurs in the COPD processes. Next, we found that 318 genes showed significantly differential expression after the treatment with the RRL (fold change value >2, adjust *P* < 0.05), of which 193 genes were upregulated, and 125 genes were downregulated in the treatment group (Figure 12(b)). The heatmap shows the top 30 genes (Figure 12(d)). GO analysis showed a similar enrichment of functions related to drug-response, inflammatory response, immune response, etc. The inflammatory response-related genes included IL-18, IL-10, IL-4R, IL-1B, and TNF-*α*, along with pathways, such as fibrosis-related ERK1/2 pathway and apoptosis (Figure 12(f)). Thus, the RRL attenuated CS and LPS-induced COPD via inhibition of inflammation, antifibrosis, and antiapoptosis.

### 3.10. The Effect of RRL on ERK1/2 Signaling, Smad3, and Apoptosis of CS and LPS-Induced COPD in Rats

**Figure 13:**
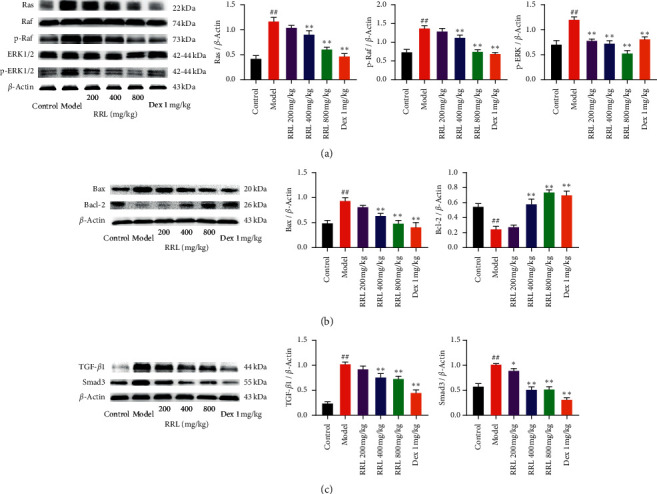
The effect of RRL on ERK1/2 signaling, Smad3, and apoptosis of CS and LPS-induced COPD in rats. (a) Statistical analysis of Ras, p-Raf, and p-ERK1/2 protein expression levels. (b) Statistical analysis of Bax and Bcl-2 protein expression levels. (c) Statistical analysis of TGF-*β*1 and Smad3 protein expression levels. Values are expressed as the mean ± SD (*n* = 3); ^##^*P* < 0.01 vs. the control group; ^*∗*^*P* < 0.05 and ^*∗∗*^*P* < 0.01 vs. the model group.

Based on the results of RNAseq, we hypothesized that ERK1/2 and TGF*β*1/Smad3 signaling pathways, as well as apoptosis, were involved in inducing the protective effects of RRL against CS and LPS-induced COPD. ERK1/2 and TGF*β*1/Smad3 are two key signaling pathways that are involved in pulmonary fibrosis. The results demonstrated that CS and LPS induced the upregulation of Bax, Ras, p-Raf, TGF-*β*1, p-ERK1/2, and Smad3 along with the downregulation of Bcl-2 (Figure 13), which activated the ERK1/2, TGF*β*1-Smad3, and apoptosis signaling pathways. The RRL could reverse these effects, thus protecting rats against CS and LPS-induced COPD by inhibiting the ERK1/2, Smad3, and apoptosis signaling pathways.

### 3.11. The Effect of RRL on NF-*κ*B, Nrf2, and Fibrosis-Related Signaling of CS and LPS-Induced COPD in Rats

**Figure 14:**
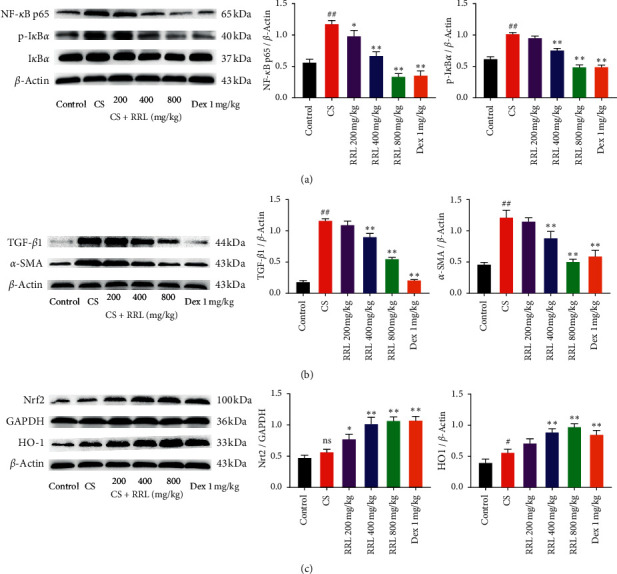
The effect of RRL on NF-*κ*B, Nrf2, and fibrosis-related signaling of CS and LPS-induced COPD in rats. (a) Statistical analysis of NF-*κ*B P65 and p-I*κ*B*α* protein expression levels. (b) Statistical analysis of TGF-*β*1 and *α*-SMA protein expression levels. (c) Statistical analysis of Nrf2 and HO-1 protein expression levels. Values are expressed as the mean ± SD (*n* = 3); ^ns^*P* > 0.05 and ^##^*P* < 0.01 vs. the control group; ^*∗*^*P* < 0.05 and ^*∗∗*^*P* < 0.01 vs. the model group.

To investigate the relationship between the effect of RRL on COPD model rats' lung inflammation, oxidative stress, and pulmonary fibrosis and the inflammation inhibition, antioxidation, and antifibrosis pathways, western blot was performed on the lung tissues. The results demonstrated that NF-*κ*B p65, p-I*κ*B*α*, TGF-*β*1, and *α*-SMA were significantly upregulated and Nrf2 and HO-1 were significantly downregulated in model rats of COPD, indicating the activation of NF-*κ*B, Nrf2, and fibrosis-related signaling pathways. Compared with the model group, the COPD model rats treated with RRL could inhibit the expression of NF-*κ*B p65, p-I*κ*B*α*, TGF-*β*1, and *α*-SMA together with upregulation of Nrf2 and HO-1(Figure 14). The results demonstrated RRL could alleviate pulmonary inflammation, oxidative stress, and pulmonary fibrosis of COPD model rats.

## 4. Discussion

The pathogenesis of COPD is related to the inflammation caused by harmful gases and particles; however, it is still unclear. Generally, smoking is considered to be a major cause of COPD [28]. The oxidants in CS can directly damage cells and tissues, destroying the defense mechanism and causing inflammation in the lungs. Thus, inflammation and oxidative stress are related to the development of COPD [29]. LPS contributes to induce emphysema in a shorter period of time than pure CS exposure (couple of months vs. 6–8 months) [30]. Here, we developed a rat model of COPD by smoke inhalation and LPS intratracheal instillation and investigated the effects of the RRL in this disease. The results of H&E and Masson staining examinations showed that CS and LPS caused serious damage to the alveolar structure, significant thickening of the alveolar septum, and infiltration of large amounts of inflammatory cells. We also found a considerable higher number of collagen fibers in the pulmonary interstitial and alveolar septum. These results demonstrated a successful induction of COPD by smoke inhalation and LPS intratracheal instillation. After treatment with the RRL, we observed a considerable reduction in the degree of inflammation and fibrosis in lung tissues of COPD rats. These experimental data indicated towards the therapeutic effect of the RRL in CS and LPS-induced COPD in rats.

Recent findings have confirmed that the NF-*κ*B pathway is involved in the development of COPD. NF-*κ*B is a heterodimer containing p65 and p50 subunits, which play a crucial role in proinflammatory signaling pathways [31, 32]. Under normal conditions, inactive NF-*κ*B binds to I*κ*B*α* in the cytoplasm, resulting in its activation by selective degradation of I*κ*B*α* through selective ubiquitination [33]. A large number of inflammatory mediators, such as chemokines and cytokines, are released by the activation of the NF-*κ*B pathway [34]. Particularly, large numbers of macrophages, neutrophils, and lymphocytes are produced, which are vital for the development of COPD since they release proinflammatory cytokines, such as IL-6, IL-8, and TNF-*α* [35, 36]. Here, we observed a substantially higher inflammatory cell count in BALF in CS and LPS-induced COPD in rats; however, the RRL considerably depleted the inflammatory cell count in BALF as well as the levels of proinflammatory factors, such as IL-6, IL-8, and TNF-*α*. Additionally, we found that the RRL exhibited a dose-dependent inhibition of CS and LPS-induced levels of p-I*κ*B*α* and NF-*κ*B p65 protein expression. These results indicated that RRL exerted a protective effect against CS and LPS-induced lung damage by inhibiting the inflammatory response.

A study showed that the ethanol extract of RRL had strong antioxidant activity, which was considered to be a strong antioxidant [13]. The main compounds of RRL ethanol extract were increased after purification with macroporous resin; we used the macroporous resin ethanol extract of RRL to investigate its protective effect against oxidative damage in the lung tissue of COPD rats. Previous experiments have shown that high levels of oxidative stress are found in COPD patients, which exceeds the ability of antioxidants, leading to oxidative imbalance and lung tissue damage. Additionally, oxidative stress can also limit the airflow in the bronchi [37–39]. Long-term exposure to CS reduces intracellular glutathione, impairing the ability of alveolar macrophages to clear the bacteria or dead cells [40]. Superoxide dismutase is the only family of enzymes with antisuperoxide free radical activity [41]. The concentration of malondialdehyde reflects the antioxidative capacity of the body, which, in turn, decides the lipid peroxidation rate and intensity of the body, and also indirectly reflects the degree of tissue peroxidation damage [42]. Nrf2 is a vital transcription factor, which regulates cellular oxidative stress [43]. When activated by toxic and harmful substances in the body, Nrf2 translocates into the nucleus to combine with the antioxidant reaction element (ARE) to form the Nrf2-ARE signaling pathway, which regulates the downstream antioxidant proteins, such as oxidases and phase II detoxifying enzymes [44, 45]. Studies have shown that Nrf2 regulates the oxidative stress protein HO-1, which maintains cellular redox homeostasis and reduces severe oxidative damage [46]. Here, we found that the RRL depleted the concentration of MDA, substantially elevated the activity of SOD and GSH-Px, and enhanced Nrf2 and HO-1 protein levels in the nucleus in CS and LPS-induced COPD in rats. Thus, the RRL exerted therapeutic effects on oxidative damage in lung tissue of COPD rats.

Prolonged exposure to smoke causes the development of pulmonary fibrosis in patients with COPD. Sustained stimulation of pathogenic factors causes the proliferation of fibroblasts (FB), transforming them into myofibroblasts (MFB), and the MFB secretes substantial amounts of extracellular matrix components (ECM), which leads to pulmonary fibrosis [47]. *α*-SMA is mainly expressed in all smooth muscle cells and myofibroblasts in the lung, which indirectly respond to the proliferation of smooth muscle cells and promote pulmonary vascular remodeling [48]. Studies have shown that TGF-*β*1 participates in airway remodeling and irreversible obstruction by inducing fibroblast proliferation, increased synthesis of collagen fibers, and extracellular matrix [49]. Phosphorylation of receptor-regulated SMADs (R-Smads), which are Smad 2/3 after the binding of TGF-*β* type I and type II receptor to TGF-*β*1, indicates the activation of TGF-*β*/Smad signaling pathway. Common Smad (co-Smad) further forms heterodimer with phosphorylated Smad 2/3 and transfers to the nucleus to mediate downstream gene expression, which results in fibrosis [50].Our results showed that the RRL reduced the degree of pulmonary fibrosis and the destruction of the alveolar structure in CS and LPS-induced COPD in rats. Simultaneously, it also reduced *α*-SMA, TGF-*β*1, and Smad3 levels in the lung tissue, thus indicating towards its therapeutic effect on the pulmonary injury of CS and LPS-induced COPD in rats.

Additionally, the results of RNAseq indicated that ERK1/2, TGF*β*1-Smad3, and apoptosis signaling pathways were involved in the protective effects of the RRL against CS and LPS-induced COPD. Many studies have reported that activation of ERK1/2 could effectively reduce the oxidative stress by controlling the expression of different genes. At the same time, the increase of oxidative stress will in turn produce more reactive oxygen species (ROS), thereby inhibiting the phosphorylation of ERK1/2 [51]. Some studies mentioned that ERK signaling pathway inhibitors are related to the weakening of NF-*κ*B signaling, and phosphorylation of ERK1/2 could trigger the NF-*κ*B signaling cascade [52–54]. In order to further verify that the antioxidation, anti-inflammatory, and antifibrosis effects of RRL on COPD model rats, we examined the expression of related proteins in lung tissue, and the results showed that the RRL could inhibit the upregulation of Ras, p-Raf, p-ERK1/2, Bax, TGF-*β*1, Smad3, NF-*κ*B p65, and p-I*κ*B*α* together with downregulation of Bcl-2 Nrf2 and HO-1. Thus, the results indicate that RRL could attenuate CS and LPS-induced COPD by inflammation inhibition and antioxidant and antifibrosis pathways.

## 5. Conclusion

In summary, this study confirmed that the RRL exhibited protection effects against CS and LPS-induced COPD in rats, and the potential mechanism involved the inhibition of the inflammatory response, enhancing its antioxidative capacity and suppressing the ERK1/2 signaling pathway. Interestingly, we found that COPD rats showed equivalent therapeutic effects after RRL (800 mg/kg) and Dex (1 mg/kg) were administered. Dex is an effective and long-acting glucocorticoid, and glucocorticoid is the first choice for the clinical treatment of COPD, further confirming that RRL plays an important role in alleviating lung inflammation and fibrosis in COPD rats. This study provides new insights into the mechanism of action of the RRL in COPD treatment.

## Figures and Tables

**Table 1:** Regressive equations, correlation coefficients, linear ranges, and contents of compounds found in the RRL.

No.	Comd.	Calibration equation	*R* ^2^	Linear range (*μ*g)	RRL (%)^a^
1	Benzyl-*β*-D-glucopyranoside	*y* = 169446.89 *x* − 6459.69	0.9966	0.345∼7.59	9.24 ± 0.12
2	Rosavin	*Y* = 2667811.94 *x* + 69388.59	0.9991	0.312∼6.864	7.12 ± 0.09
3	Rosin	*Y* = 2215862.42 *x* + 40138.65	0.9989	0.1155∼2.541	2.72 ± 0.06
4	Rosiridin	*y* = 248630.00 *x* + 2913.56	0.9984	0.96∼21.12	29.23 ± 0.16
5	Gallic acid	*y* = 18922835.48 x - 27262.09	0.9983	0.0238∼0.5236	0.64 ± 0.04
6	Rosarin	*y* = 2666581.10 *x* + 31253.97	0.9989	0.097∼2.134	2.25 ± 0.07
7	Rhodiocyanoside A	*y* = 2106103.26 *x* + 3212.22	0.9989	0.06708∼1.47576	1.78 ± 0.11
8	Salidroside	*y* = 3443149.16 *x* + 21142.66	0.9988	0.12194∼2.68268	3.00 ± 0.13

^a^Values are expressed as mean ± standard deviation of three determinations.

**Table 2:** The effect of RRL on the respiratory improvement of CS and LPS-induced COPD in rats.

Group	The lung organ coefficient	The lung wet/dry weight ratio
Control	1.474 ± 0.02979	0.4481 ± 0.02956
Model	2.166 ± 0.0365^#^	0.7365 ± 0.02847^##^
RRL 200 mg/kg	1.942 ± 0.05976	0.5977 ± 0.009329
RRL 400 mg/kg	1.915 ± 0.0433	0.575 ± 0.02703
RRL 800 mg/kg	1.703 ± 0.07538^*∗∗*^	0.4998 ± 0.01312^*∗*^
Dex 1 mg/kg	1.608 ± 0.03422^*∗*^	0.53 ± 0.02465^*∗∗*^

Values are expressed as the mean ± SD; ^#^*P* < 0.05 vs. the control group; ^##^*P* < 0.01 vs. the control group; ^*∗*^*P* < 0.05 and ^*∗∗*^*P* < 0.01 vs. the model group.

## Data Availability

All data and materials used in the present study are available from the corresponding author upon reasonable request.
